# Supplementary material for: Acute Kidney Injury Following Admission with Acute Coronary Syndrome: The Role of Diabetes Mellitus
Source: J Clin Med. 2021 Oct 25;10(21):4931. doi: 10.3390/jcm10214931 (PMC8584470; doi:10.3390/jcm10214931)
Supplement: Supplementary file 1 [file jcm-10-04931-s001.zip › jcm-1389985-supplementary.pdf]

Table S1. Baseline Characteristics by AKI.

	AKI	No-AKI	*P*
n	1,016	15,863	
**Baseline characteristics and demographics**			
Age, years (median [IQR])	75 67, 82]	63 [54, 73]	<0.001
Gender (male)	710 (69.9)	12,328 (77.7)	<0.001
Higher education/ academic	62 (26.5)	1393 (29.6)	0.35
Marital status: married	323 (65.5)	6628 (79.2)	<0.001
Dyslipidaemia	611 (60.4)	10357 (65.5)	0.001
Hypertension	758 (74.7)	9277 (58.6)	<0.001
Current smokers	215 (21.4)	6088 (38.6)	<0.001
Diabetes mellitus	513 (50.5)	5587 (35.2)	<0.001
Family history of CAD	126 (13.8)	3946 (26.7)	<0.001
BMI (kg/m2), (median [IQR])	26.6 [24.2, 29.4]	27 [24.6, 30.1]	0.008
Prior MI	405 (40)	4870 (30.7)	<0.001
Prior CABG	157 (15.5)	1498 (9.5)	<0.001
Prior PCI	287 (28.4)	4478 (28.3)	0.97
Chronic renal failure	458 (45.1)	1374 (8.7)	<0.001
PVD	205 (20.2)	1188 (7.5)	<0.001
s/p CVA/TIA	146 (14.4)	1227 (7.8)	<0.001
History of CHF	233 (23)	1130 (7.1)	<0.001
Grace score>140	244 (46.9)	1207 (13.2)	<0.001
Earliest creatinine (mg/dL) (median [IQR])	1.76 [1.4, 2.4]	1 [0.84, 1.2]	<0.001
**Medical therapy prior to admission**			
Aspirin	477 (56.2)	6544 (47.3)	<0.001
Clopidogrel	82 (9.7)	1274 (9.4)	0.772
ACE-I	209 (38.1)	2954 (30.9)	<0.001
ARB	98 (18.1)	1118 (12.1)	<0.001
Beta blockers	388 (46.3)	4947 (36.7)	<0.001
Statins	400 (49.0)	6201 (47.3)	0.355
Calcium channel blockers	271 (32.9)	2718 (20.8)	<0.001
Nitrates	176 (21.6)	1355 (10.5)	<0.001
Hypoglycaemic agents	262 (30.3)	3226 (22.7)	<0.001
Diuretics	261 (36.2)	1805 (16.2)	<0.001
Coronary angiography and PCI			
PCI	481 (47.3)	9827 (61.9)	<0.001
STEMI	458 (45.1)	7201 (45.4)	0.85
Coronary angiography	486 (69.4)	10,942 (88.2)	<0.001
**Medical therapy upon discharge**			
Aspirin	729 (81.7)	14,828 (95.1)	<0.001
P2Y12	499 (56.2)	11,427 (73.8)	<0.001
Statins	557 (62.9)	13,389 (86.5)	<0.001
ACE-I/ARB	478 (52.4)	11,551 (75.5)	<0.001
Beta blockers	547 (61.3)	12,326 (80.9)	<0.001

CHF: congestive heart failure; **CAD**: coronary artery disease; **BMI**: body mass index; **MI**: myocardial infarction; **TIA**: transient ischemic attack; **CVA**: cerebrovascular attack; **ACE**: angiotensin converting enzyme; **ARB**: angiotensin receptor blockers, **PVD**: peripheral vascular (arterial) disease; **PCI**: percutaneous coronary interventional; **CABG**: coronary artery bypass graft surgery.

Table S2. multivariable model for prediction of AKI among ACS patients.

variable	OR (95% CI)	p
Diabetes mellitus	1.40	**$p < 0.001$**
	(1.21, 1.61)	
Age (per 5 years)	1.25	**$p < 0.001$**
	(1.20, 1.29)	
Gender (male)	0.93	$p = 0.39$
	(0.80, 1.09)	
Dyslipidaemia	0.67	**$p < 0.001$**
	(0.57, 0.77)	
Hypertension	1.07	$p = 0.43$
	(0.91, 1.27)	
Current smokers	0.95	$p = 0.55$
	(0.79, 1.13)	
Family history of CAD	0.84	$p = 0.10$
	(0.68, 1.03)	
Prior CABG	0.94	$p = 0.54$
	(0.76, 1.15)	
Prior PCI	0.77	**$p = 0.002$**
	(0.65, 0.90)	
Chronic renal failure	4.91	**$p < 0.001$**
	(4.20, 5.74)	
Prior PVD	1.55	**$p < 0.001$**
	(1.29, 1.87)	
Prior CVA/TIA	0.99	$p = 0.94$
	(0.81, 1.21)	
History of CHF	1.57	**$p < 0.001$**
	(1.31, 1.89)	

CHF: congestive heart failure; **CAD**: coronary artery disease; **TIA**: transient ischemic attack; **CVA**: cerebrovascular attack; **PVD**: peripheral vascular (arterial) disease**; PCI**: percutaneous coronary interventional; **CABG**: coronary artery bypass graft surgery.

Table S3. Multivariable Model for Prediction of 1-year All-cause Mortality following ACS.

variable	OR (95% CI)	p
AKI	4	**$p < 0.001$**
	(3.39, 4.73)	
Diabetes mellitus	1.47	**$p < 0.001$**
	(1.31, 1.66)	
Age (per 5 years)	1.3	**$p < 0.001$**
	(0.74, 0.93)	
Gender (male)	0.83	**$p < 0.001$**
	(0.80, 1.09)	
Dyslipidaemia	0.63	**$p < 0.001$**
	(0.56, 0.70)	
Hypertension	1.01	$p = 0.94$
	(0.89, 1.13)	
Current smokers	1.12	$p = 0.1$
	(0.98, 1.28)	
Family history of CAD	0.69	**$p < 0.001$**
	(0.58, 0.82)	
Prior CABG	0.96	$p = 0.59$

	(0.82, 1.12)	
Prior PCI	0.82	***p* = 0.002**
	(0.65, 0.90)	
Chronic renal failure	1.24	***p* = 0.001**
	(1.09, 1.41)	
Prior PVD	1.54	***p* < 0.001**
	(1.35, 1.76)	
Prior CVA/TIA	1.38	***p* < 0.001**
	(1.20, 1.58)	
History of CHF	1.66	***p* < 0.001**
	(1.45, 1.89)	
Interaction AKI* DM	0.83	***p* = 0.1**
	(0.66, 1.03)	

CHF: congestive heart failure; **CAD**: coronary artery disease; **TIA**: transient ischemic attack; **CVA**: cerebrovascular attack; **PVD**: peripheral vascular (arterial) disease**; PCI**: percutaneous coronary interventional; **CABG**: coronary artery bypass graft surgery.

Table S4. Baseline Characteristics by DM.

	**No-DM**	**DM**	**P**
n	10,779	6,100	
**Baseline characteristics and demographics**			
Age, years (median [IQR])	61.00 [52.00, 73.00]	66.00 [58.00, 75.00]	<0.001
Gender (male)	8626 (80.0)	4412 (72.3)	<0.001
Higher education/ academic	989 (33.0)	466 (24.0)	<0.001
Marital status: married	4292 (78.5)	2659 (78.3)	0.809
Dyslipidaemia	6299 (58.6)	4669 (76.8)	<0.001
Hypertension	5428 (50.4)	4607 (75.7)	<0.001
Current smokers	4501 (41.9)	1802 (29.8)	<0.001
Family history of CAD	2807 (27.6)	1265 (23.1)	<0.001
BMI (kg/m2), (median [IQR])	26.56 [24.28, 29.41]	27.74 [25.25, 31.14]	<0.001
Prior MI	2825 (26.2)	2450 (40.3)	<0.001
Prior CABG	785 (7.3)	870 (14.3)	<0.001
Prior PCI	2479 (23.0)	2286 (37.6)	<0.001
Chronic renal failure	766 (7.1)	1066 (17.5)	<0.001
PVD	596 (5.5)	797 (13.1)	<0.001
s/p CVA/TIA	656 (6.1)	717 (11.8)	<0.001
History of CHF	583 (5.4)	780 (12.8)	<0.001
Grace score>140	692 (11.5)	759 (21.0)	<0.001
Earliest creatinine (mg/dL) (median [IQR])	1.00 [0.86, 1.19]	1.03 [0.85, 1.38]	<0.001
**Medical therapy prior to admission**			
Aspirin	3623 (38.8)	3398 (63.6)	<0.001
Clopidogrel	622 (6.8)	734 (14.0)	<0.001
ACE-I	1468 (23.4)	1695 (44.2)	<0.001
ARB	524 (8.6)	692 (18.7)	<0.001
Beta blockers	2796 (30.8)	2539 (48.4)	<0.001
Statins	3394 (38.7)	3207 (62.3)	<0.001
Calcium channel blockers	1484 (16.8)	1505 (29.7)	<0.001
Nitrates	748 (8.5)	783 (15.7)	<0.001

Hypoglycaemic agents	36 (0.4)	3452 (62.5)	<0.001
Diuretics	919 (12.4)	1147 (26.1)	<0.001
**Coronary angiography and PCI**			
PCI	6868 (63.7)	3440 (56.4)	<0.001
STEMI	2813 (63.1)	1127 (55.1)	<0.001
Coronary angiography	7298 (89.0)	4130 (84.4)	<0.001

CHF: congestive heart failure; **CAD**: coronary artery disease; **BMI**: body mass index; **MI**: myocardial infarction; **TIA**: transient ischemic attack; **CVA**: cerebrovascular attack; **ACE**: angiotensin converting enzyme; **ARB**: angiotensin receptor blockers, **PVD**: peripheral vascular (arterial) disease; **PCI**: percutaneous coronary interventional; **CABG**: coronary artery bypass graft surgery.

Figure S1. Multivariable Analysis for Prediction of 1-year Mortality according to DM Status.

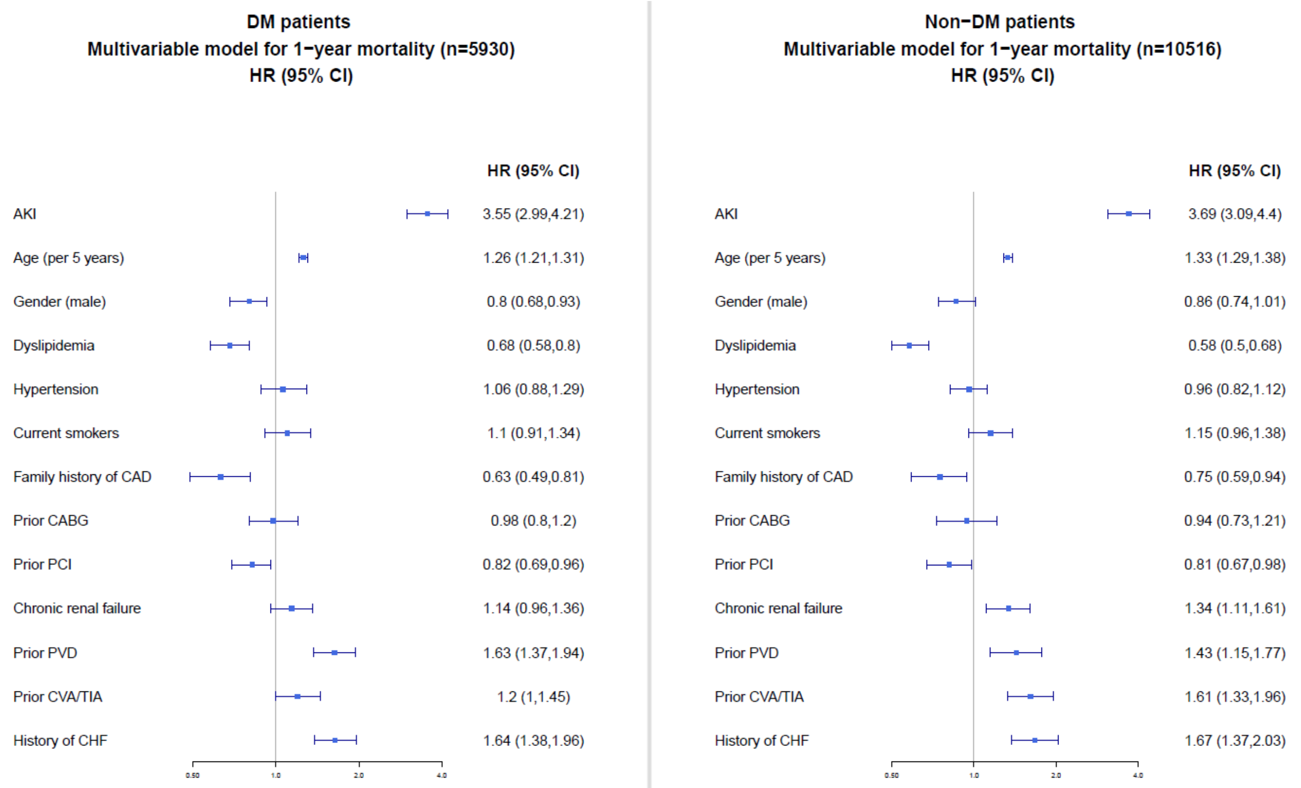

CHF: congestive heart failure; **CAD**: coronary artery disease; **TIA**: transient ischemic attack; **CVA**: cerebrovascular attack; **PVD**: peripheral vascular (arterial) disease**; PCI**: percutaneous coronary interventional; **CABG**: coronary artery bypass graft surgery.
